# Tocotrienol Rich Palm Oil Extract Is More Effective Than Pure Tocotrienols at Improving Endothelium-Dependent Relaxation in the Presence of Oxidative Stress

**DOI:** 10.1155/2015/150829

**Published:** 2015-05-17

**Authors:** Saher F. Ali, Owen L. Woodman

**Affiliations:** School of Medical Sciences, Health Innovations Research Institute, RMIT University, Bundoora, VIC 3083, Australia

## Abstract

Oxidative endothelial dysfunction is a critical initiator of vascular disease. Vitamin E is an effective antioxidant but attempts to use it to treat vascular disorders have been disappointing. This study investigated whether tocotrienols, the less abundant components of vitamin E compared to tocopherols, might be more effective at preserving endothelial function. Superoxide generated by hypoxanthine/xanthine oxidase or rat aorta was measured using lucigenin-enhanced chemiluminescence. The effect of *α*-tocopherol, *α*-, *δ*-, and *γ*-tocotrienols and a tocotrienol rich palm oil extract (tocomin) on levels of superoxide was assessed. Endothelial function in rat aorta was assessed in the presence of the auto-oxidant pyrogallol. Whilst all of the compounds displayed antioxidant activity, the tocotrienols were more effective when superoxide was produced by hypoxanthine/xanthine oxidase whereas tocomin and *α*-tocopherol were more effective in the isolated aorta. Tocomin and *α*-tocopherol restored endothelial function in the presence of oxidant stress but *α*-, *δ*-, and *γ*-tocotrienols were ineffective. The protective effect of tocomin was replicated when the tocotrienols were present with, but not without, *α*-tocopherol. Tocotrienol rich tocomin is more effective than *α*-tocopherol at reducing oxidative stress and restoring endothelium-dependent relaxation in rat aortae and although *α*-, *δ*-, and *γ*-tocotrienols effectively scavenged superoxide, they did not improve endothelial function.

## 1. Introduction

Vitamin E, in addition to the four isoforms of tocopherol, contains four isoforms of tocotrienol. While there has been extensive investigation of the biological activity of the tocopherols, there has been much less attention paid to the tocotrienols. There is, however, emerging evidence that the tocotrienols have molecular targets distinct from those of the tocopherols that may result in new therapeutic opportunities [1]. There are now a number of studies demonstrating cardioprotective actions of tocotrienols. For example, *γ*-tocotrienol is known to inhibit HMG-CoA reductase and therefore to decrease cholesterol synthesis [2]. Further, extracts of palm oil, a rich source of tocotrienols, have been demonstrated to activate the NO-cGMP pathway and, as a consequence, to decrease myocardial reperfusion injury [3] perhaps due to scavenging of peroxynitrite [4]. The antioxidant activity of tocotrienols may also contribute to protective actions in the vasculature [1]. For example, Newaz et al. [5] demonstrated that treatment of spontaneously hypertensive rats with *γ*-tocotrienol increased NOS activity and lowered arterial pressure, and *γ*-tocotrienol has also been shown to reduce oxidative stress and inflammation in rats with streptozotocin- (STZ-) induced diabetes [6]. Further Norsidah et al. [7] reported that a palm oil extract rich in tocotrienols, when orally administered to rats with hyperhomocysteinemia, reduced aortic oxidative stress and increased the plasma level of NO metabolites [7]. In addition, Muharis et al. [8] recently demonstrated that a palm oil fraction rich in tocotrienols restored endothelium-dependent relaxation in arteries in rats with STZ-induced type 1 diabetes but it is not clear whether this may have been consequent to a lowering of glucose levels as reported by Budin et al. [9]. There is evidence that the beneficial vascular effects of tocotrienols may extend to man given the report that 2-month treatment with tocotrienols improves pulse wave velocity in healthy males [10].

The mechanism(s) of the beneficial effects of tocotrienols have not been well investigated nor, to the best of our knowledge, has there been any examination of the vascular actions of individual tocotrienol isomers. Therefore, the aims of this study were to compare the antioxidant activity of *α*-, *δ*-, and *γ*-tocotrienols with *α*-tocopherol and tocomin, a palm oil extract rich in tocotrienols (tocotrienol rich fraction: 40%, and palm olein: 38%) but also containing some *α*-tocopherol (11%). Given the antioxidant activity of these compounds we were further interested to investigate their capacity to protect NO-mediated vascular relaxation as an indication of whether they may be effective in preventing endothelial dysfunction in vascular diseases involving oxidant stress, for example, as a result of diabetes [11, 12]. It has been reported that tocotrienols are incorporated into cellular membranes more rapidly than tocopherol [13–15] and that this may contribute to greater antioxidant efficacy. We therefore hypothesized that the tocotrienols would more effectively preserve endothelium-dependent relaxation in the presence of oxidative stress.

## 2. Materials and Methods

### 2.1. Animals

Male Wistar rats 6–8 weeks of age (240–280 g) (Animal Resource Centre, Perth, WA, Australia) were used in the study. All procedures were approved by the Animal Experimentation Ethics Committee of RMIT University and conformed to the National Health and Medical Research Council of Australia code of practice for the care and use of animals for scientific purposes (AEC approval numbers 1309 and 1211).

### 2.2. Isolation of Aorta

The rats were killed by CO_2_ inhalation, followed by decapitation. The thoracic aorta was isolated and immediately placed in ice-cold Krebs bicarbonate solution (118 mM NaCl, 4.7 mM KCl, 1.18 mM MgSO_4_, 1.2 mM KH_2_PO_4_, 25 mM NaHCO_3_, 11.1 mM D-glucose, and 1.6 mM CaCl_2_). The aorta was then cleared of fat and connective tissue and cut into 2-3 mm long segments. The aortic rings were mounted between two stainless steel wires, one of which was linked to an isometric force transducer (model FT03, Grass Medical Instruments, Quincy, MA, USA) connected to a MacLab/8 (model MKIII, AD Instrument Co., Sydney, Australia), and the other end anchored to a glass rod submerged in a standard 10 mL organ bath. The organ bath was filled with Krebs-bicarbonate solution. The bath medium was maintained at 37°C, pH 7.4, and continuously aerated with 95% O_2_ and 5% CO_2_. Aortic rings were equilibrated for 45 minutes at a resting tension of 1 g and then were precontracted with an isotonic, high potassium physiological salt solution (KPSS, 122.7 mM KCl, in which K^+^ ions replaced Na^+^ ions in the solution) for 20 minutes to achieve maximal contraction. After reequilibration, the rings were submaximally contracted with phenylephrine (PE, 0.01–0.3 M) and endothelial integrity was tested by a single concentration of acetylcholine (ACh, 10^−5 ^M). Where relaxation was greater than 80% of the precontraction, the endothelium was considered to be intact and the aortic ring was included in the study. Some additional segments of the thoracic aortae were used to measure superoxide production.

### 2.3. Superoxide Generation Using Hypoxanthine/Xanthine Oxidase

Superoxide production was also measured by lucigenin enhanced chemiluminescence using hypoxanthine plus xanthine oxidase as a generator of oxygen radicals. Krebs-HEPES buffer (300 *μ*L) containing lucigenin (5 mmol/L) and appropriate treatments were placed into a 96-well OptiPlate, followed by the addition of 1 unit/mL xanthine oxidase. A background reading was performed after which hypoxanthine (10^−4 ^M) was added to all wells and superoxide production was measured. Superoxide inhibition was quantified by subtracting the superoxide reading from the background reading and expressing them as a percentage of the counts in the presence of the control.

### 2.4. Superoxide Generation by Aorta

Superoxide production in the thoracic aorta was measured using lucigenin enhanced chemiluminescence based on methods described by Leo et al. [11] with the following modification. The thoracic aorta was isolated, cleared of fat and connective tissue, and cut into 2-3 mm long segments in Krebs-HEPES buffer (composition (mM): NaCl 99.90, KCl 4.7, KH_2_PO_4_ 1.0, MgSO_4_·7H_2_O 1.2, D-glucose 11.0, NaHCO_3_ 25.0, CaCl_2_·2H_2_O 2.5, Na HEPES 20.0, pH 7.4). Aortic ring segments were incubated at 37°C for 45 min in Krebs-HEPES buffer in the presence of NADPH (100 mmol/L) as a substrate for NADPH oxidase and either alone or in the presence of tocomin, *α*-tocopherol, or *α*-, *δ*-, or *γ*-tocotrienol. In addition superoxide was measured in the presence of diphenylene iodonium (DPI, 5 mmol/L), a flavoprotein inhibitor that inhibits NADPH oxidase, as a positive control. 300 *μ*L of Krebs-HEPES buffer containing lucigenin (5 mmol/L) and the appropriate treatments were placed into a 96-well OptiPlate, and superoxide production was measured and quantified.

### 2.5. Vascular Function Experiments

Cumulative concentration response curves to ACh (0.1 nM–10 mM) and sodium nitroprusside (SNP, 0.1 nM–10 mM) were determined using aortic rings contracted with phenylephrine (10^−8^ to 10^−7 ^M) to 40%–60% of maximal contraction. Oxidative stress was generated by the addition of pyrogallol (30 *μ*M) as previously described [16]. Pyrogallol is well established to auto-oxidise to generate superoxide which then impairs endothelium-dependent relaxation by inactivating NO [17]. Responses to ACh and SNP were also tested in the presence of pyrogallol by exposing the aortae for 20 minutes to tocotrienol rich tocomin (10^−6^–10^−4 ^mg/mL), *α*-tocopherol (10^−4^–10^−2 ^mg/mL), or tocotrienol isomers (*α*-, *δ*-, or *γ*-tocotrienol 10^−3^–10^−1^ mg/mL) to determine the effect of tocomin, *α*-tocopherol or *α*-, *δ*-, or *γ*-tocotrienol on endothelium-dependent and -independent relaxation in the presence of oxidative stress and if there is any potency difference between tocomin, *α*-tocopherol, and *α*-, *δ*-, or *γ*-tocotrienol.

Responses to ACh and SNP were also tested in the presence of pyrogallol plus various combinations of *α*-tocopherol and tocotrienol isomers to replicate tocomin (10% *δ*-tocotrienol: 20% *α*-tocotrienol: 50% *γ*-tocotrienol: 20% *α*-tocopherol) and other tocotrienol combinations (*α*+*γ*)-tocotrienols and (*α*+*δ*+*γ*)-tocotrienols at a concentration of 10^−4^ mg/mL. These experiments were conducted to determine whether an interaction between *α*-tocopherol and the tocotrienols is necessary to improve endothelium-dependent relaxation in the presence of oxidative stress.

### 2.6. Reagents

All drugs were purchased from Sigma Aldrich except for acetylcholine perchlorate (BDH Chemicals, Poole, Dorset, UK), tocomin, and *α*-, *δ*-, and *γ*-tocotrienols (Carotech, Malaysia). All drugs were dissolved in distilled water, with the exception of tocomin, *α*-tocopherol, and *α*-, *δ*-, and *γ*-tocotrienols that were dissolved in 0.1% DMSO. A mixture of *α*-tocopherol and *α*-, *δ*-, and *γ*-tocotrienols which resembles tocomin was prepared as per the tocomin MSDS (10% *δ*-tocotrienol: 20% *α*-tocotrienol: 50% *γ*-tocotrienol: 20% *α*-tocopherol). Various tocotrienol combinations were also prepared using the following proportions: (*α*+*γ*)-tocotrienols (20% *α*-tocotrienol: 50% *γ*-tocotrienol: 30% DMSO) and (*α*+*δ*+*γ*)-tocotrienols (10% *δ*-tocotrienol: 20% *α*-tocotrienol: 50% *γ*-tocotrienol: 20% DMSO).

### 2.7. Statistical Analyses

All results are expressed as mean ± SEM, where *n* represents the number of animals per group. Concentration-response curves from the rat-isolated aortae were constructed and fitted to a sigmoidal curve using nonlinear regression (Graphpad Prism version 6.0, Graphpad Software, San Diego, CA, USA) to calculate the sensitivity of each agonist (pEC_50_). Maximum relaxation (*R*_max⁡_) to ACh was measured as a percentage of the precontraction to phenylephrine. Group pEC_50_ and *R*_max⁡_ values were compared using a one-way ANOVA with post hoc analysis using Sidak's test as appropriate. *p* < 0.05 was considered statistically significant.

Superoxide levels from rat aortic rings are expressed as average counts per second ± SEM normalized to dry tissue weight. Results were compared by one-way ANOVA with a post hoc Dunnett's test. *p* < 0.05 was considered statistically significant.

Results from superoxide production and antioxidant capacity using hypoxanthine/xanthine oxidase are expressed as a percentage of the counts in the presence of the control (0.1% Krebs-HEPES buffer). The level of superoxide inhibition at each concentration was compared to vehicle for each compound using 1-way ANOVA with post hoc multiple comparisons using Dunnett's test (Prism version 6.0). *p* < 0.05 was considered statistically significant.

## 3. Results

### 3.1. Superoxide Scavenging Capacity of Tocomin, *α*-Tocopherol, and *α*-, *δ*-, and *γ*-Tocotrienols Using Hypoxanthine/Xanthine Oxidase

**Figure 1:**
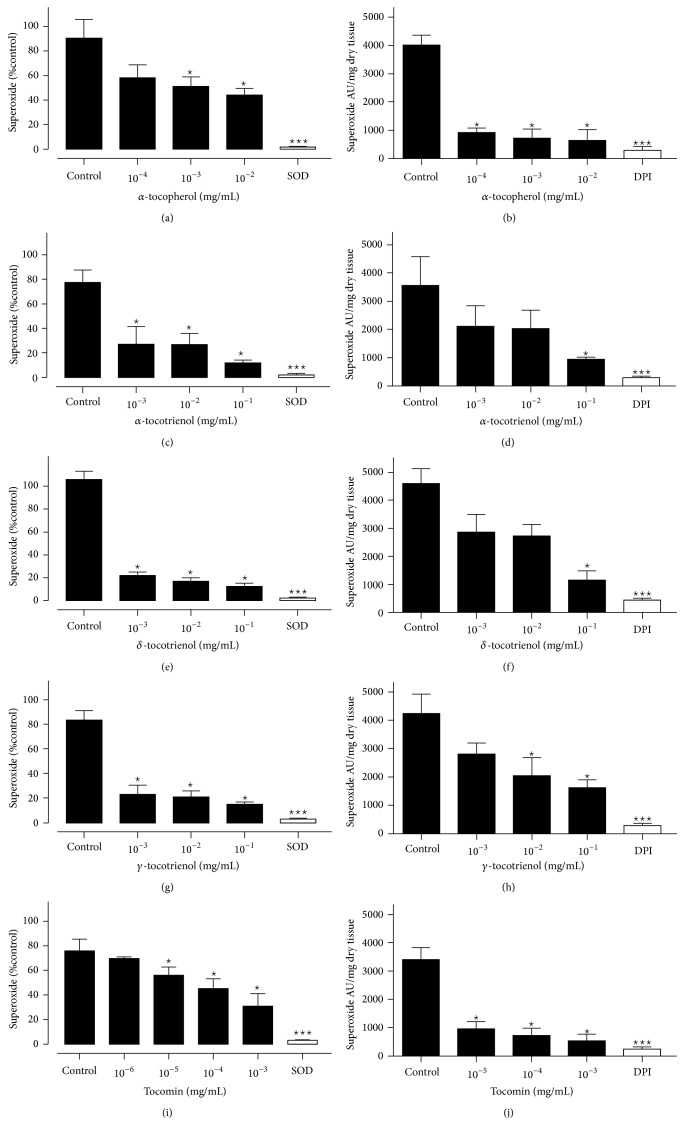
Superoxide generated by hypoxanthine (100 *μ*M)/xanthine oxidase (0.01 U/mL) or rat aorta in the presence of NADPH: tocomin ((a) and (b)), *α*-tocopherol ((c) and (d)), *α*-tocotrienol ((e) and (f)), *δ*-tocotrienol ((g) and (h)), and *γ*-tocotrienols ((i) and (j)). Data is expressed as mean ± SEM. ^*^Significantly different to control *p* < 0.05. ^***^Significantly different to control *p* < 0.001. Dunnett's multiple comparisons test.

Superoxide production induced by the presence of hypoxanthine/xanthine oxidase is shown in Figure 1. *α*-tocopherol (1A) caused an approximately 50% reduction in superoxide at a concentration of 10^−2 ^mg/mL (Figure 1(a)). At the same concentration, all of the tocotrienol isomers caused approximately 80% reductions in superoxide (Figures 1(c), 1(e), and 1(g)). Tocomin (Figure 1(i)) caused a 50% inhibition of superoxide similar to *α*-tocopherol but at a concentration 10–100 times lower.

### 3.2. Superoxide Scavenging Capacity of Tocomin, *α*-Tocopherol, and *α*-, *δ*-, and *γ*-Tocotrienol in Rat Aorta

As observed with the hypoxanthine/xanthine oxidase assay all of the compounds of interest were able to significantly reduce superoxide levels but the potency and efficacy were quite different (Figures 1(b), 1(d), 1(f), 1(h), and 1(j)). Interestingly, whereas *α*-tocopherol and tocomin produced relatively greater inhibition of aorta-derived superoxide, the tocotrienol isomers were less effective at the same concentrations in the hypoxanthine/xanthine oxidase assay. The relative potency between *α*-tocopherol and tocomin remained the same in this assay with tocomin being effective at approximately 100-fold lower concentration.

### 3.3. Vascular Function

**Figure 2:**
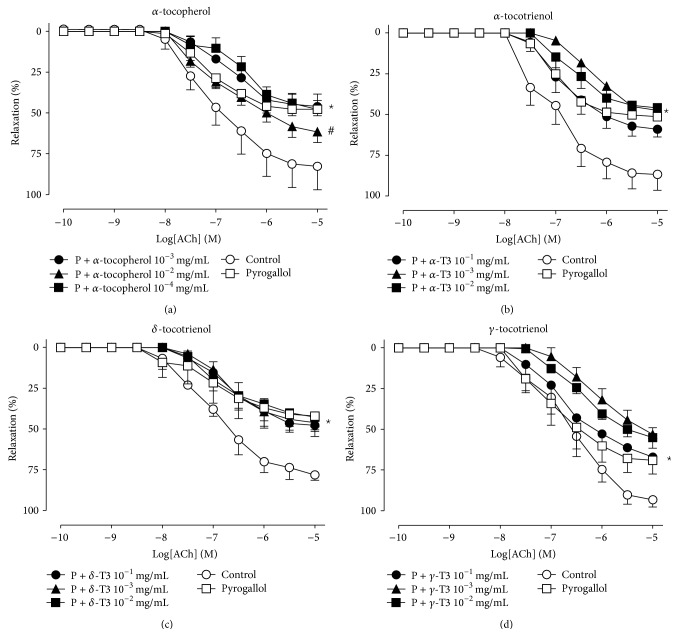
Endothelium-dependent relaxation in rat aortae in the presence of pyrogallol (P). Cumulative concentration-response curves to ACh in the absence (control) or presence of pyrogallol with varying concentrations of *α*-tocopherol (a), *α*-tocotrienol (b), *δ*-tocotrienol (c), and *γ*-tocotrienols (d). Data is expressed as mean ± SEM. ^*^Significantly different to control *p* < 0.05. ^#^Significantly different to pyrogallol *p* < 0.05 Sidak's multiple comparison test.

**Figure 3:**
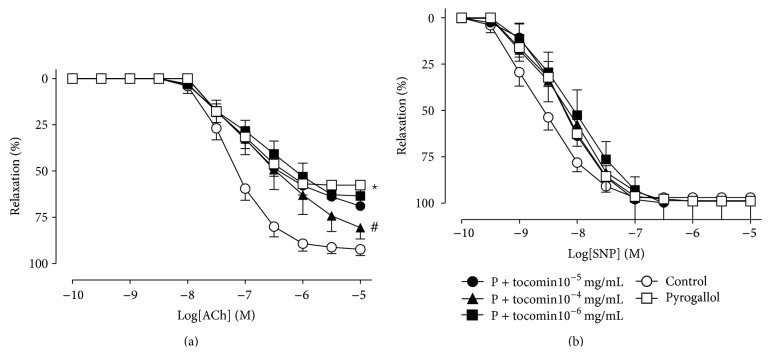
Endothelium-dependent and -independent relaxation in rat aortae in the presence of pyrogallol (P): cumulative concentration-response curves to ACh (a) and SNP (b) in the absence (control) or presence of pyrogallol with varying concentrations of tocomin. Data is expressed as mean ± SEM. ^*^Significantly different to control *p* < 0.05. ^#^Significantly different to pyrogallol *p* < 0.05 Sidak's multiple comparison test.

The effect of pyrogallol-induced oxidative stress and the acute addition of varying concentrations of *α*-tocopherol, the tocotrienols, and tocomin is shown in Figures 2 and 3. Endothelium-dependent relaxation in response to ACh was significantly inhibited in the presence of pyrogallol-induced oxidative stress with a significant decrease in *R*_max⁡_ without affecting pEC_50_ (Table 1). Both *α*-tocopherol (Figure 2(a), 10^−2 ^mg/mL) and tocomin (Figure 3(a), 10^−4 ^mg/mL) were able to significantly improve endothelium-dependent relaxation in the presence of pyrogallol; however, tocomin improved endothelium-dependent relaxation at a concentration 100 times lower compared to *α*-tocopherol (Table 1). None of the tocotrienol isomers (*α*-, *δ*-, and *γ*-tocotrienols) improved endothelium-dependent relaxation even at concentrations 100 times higher than that of *α*-tocopherol (Table 1). Endothelium-independent relaxation to SNP was not affected by pyrogallol, *α*-tocopherol, tocomin, or the tocotrienols (Table 1).

**Figure 4:**
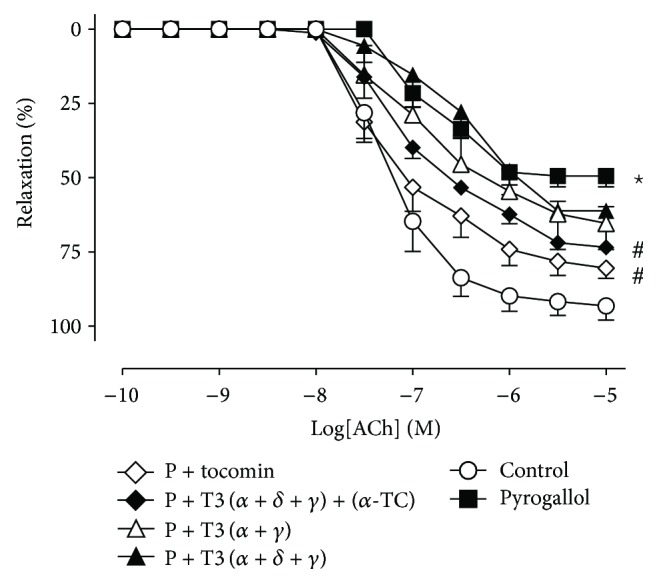
Endothelium-dependent relaxation in rat aortae in the presence of pyrogallol (P): cumulative concentration-response curves to ACh in the absence (control) or presence of pyrogallol with varying combinations of *α*-tocopherol (TC) and *α*-, *δ*-, and *γ*-tocotrienols (T3) (10^−4 ^mg/mL). Tocotrienol isomers and *α*-tocopherol were present in the proportions found in tocomin (i.e., *α*-T3- 20%, *δ*-T3 10%, *γ*-T3 50%, and *α*-TC 20%). Data is expressed as mean ± SEM. ^*^Significantly different to control *p* < 0.05. ^#^Significantly different to pyrogallol *p* < 0.05 Sidak's multiple comparison test.

The effect of pyrogallol-induced oxidative stress and the acute addition of varying combinations of *α*-tocopherol and *α*-, *δ*-, and *γ*-tocotrienols (10^−4^ mg/mL) is shown in Figure 4. Both tocomin and the mixture of (T3(*α*+*δ*+*γ*) + (*α*-TC)) (Figure 4, 10^−4^ mg/mL) significantly improved endothelium-dependent relaxation in the presence of pyrogallol (Table 2 and Figure 4). Other preparations in the absence of *α*-TC, that is, T3(*α*+*γ*) and (*α*+*δ*+*γ*)-tocotrienols (10^−4 ^mg/mL), did not improve endothelium-dependent relaxation (Figure 4 and Table 2). Endothelium-independent relaxation was not affected by the presence of pyrogallol, *α*-tocopherol, or *α*+*δ*+*γ*-tocotrienols (Table 2).

## 4. Discussion

This study demonstrated that the tocotrienol isomers were more effective at scavenging superoxide radicals produced by hypoxanthine/xanthine oxidase in comparison to those generated by isolated aortic segments in the presence of NADPH. Tocomin and *α*-tocopherol restored endothelial function in the presence of oxidative stress but *α*-, *δ*-, and *γ*-tocotrienols were ineffective. *α*-tocopherol was less effective than the tocotrienol isomers at similar concentrations when superoxide was generated by hypoxanthine/xanthine oxidase but more effective against superoxide generated by vascular tissue. Tocomin, an extract of palm oil containing predominantly tocotrienols but with some tocopherol, was effective in both assays at 100-fold lower concentrations than *α*-tocopherol. Consistent with their relatively lower antioxidant activity in isolated vascular tissue, the tocotrienol isomers failed to improve endothelium-dependent relaxation in the presence of oxidant stress. Surprisingly tocomin was the most effective compound at improving endothelium-dependent relaxation and this effect could be replicated by a mixture of *α*-tocopherol and *α*-, *δ*-, and *γ*-tocotrienols, suggesting that the tocotrienol isomers provide more effective vasoprotection when acting together in combination with *α*-tocopherol.

In the present study the antioxidant capacity of *α*-tocopherol, tocomin, and *α*-, *β*-, or *γ*-tocotrienols was examined using hypoxanthine/xanthine oxidase to generate superoxide in a tissue-free system or superoxide was produced by NADPH oxidase in segments of rat isolated aorta in the presence of NADPH. We have used both of these assays previously when testing the antioxidant activity of flavonols as a tool to predict efficacy as vasoprotectants in vascular disease [16]. Xanthine oxidase (XO) is located on blood vessel walls [18] and is an important enzyme that catalyzes the conversion of hypoxanthine to xanthine as a part of purine metabolism producing superoxide (O_2_^−^) and hydrogen peroxide as a byproduct [19]. XO induced free radical production has been implicated in the pathogenesis of diabetes related vascular complications [20]. *α*-tocopherol and *α*-, *δ*-, and *γ*-tocotrienol were able to scavenge O_2_^−^ at concentrations as low as 10^−3 ^mg/mL whereas tocotrienol rich tocomin was able to achieve the same effect at concentrations as low as 10^−5 ^mg/mL. Tocopherol and tocotrienols have been demonstrated to exert their antioxidant activity by physically quenching superoxide [21]. Our findings suggest that *α*-tocopherol is 10 times more potent than *α*-, *δ*-, and *γ*-tocotrienols at scavenging hypoxanthine induced O_2_^−^ is somewhat surprising given the report by Yoshida et al. [22] that tocopherol and tocotrienol isomers have a similar antioxidant activity when tested in homogenous solutions. A further surprising observation was that the tocotrienol isomers were less effective at scavenging superoxide derived from the aortic segments as this suggests a limited ability to access the tissue derived reactive oxygen species. This is in contrast to previous observations that tocotrienols are rapidly incorporated into cell membranes, a contributing factor to their antioxidant efficacy [13–15].

The relative antioxidant efficacy of the compounds under examination was different when aortic segments provided the source of superoxide. Tocomin, containing a mixture of tocotrienol isomers and *α*-tocopherol, was more effective than the individual isomers at reducing oxidative stress whereas in the hypoxanthine/xanthine oxidase assay the opposite situation was observed. As noted above this may indicate an increase in activity when the tocotrienol isomers are combined or perhaps there is also an interaction with *α*-tocopherol.

Our next aim was to investigate whether the compounds could effectively improve endothelium-dependent relaxation impaired by the presence of oxidative stress. Endothelium-derived nitric oxide (NO) rapidly reacts with O_2_^−^ (rate constant 2 × 10^10^ M/sec) [23], which reduces its relaxant activity. Superoxide dismutase (SOD) also reacts rapidly with O_2_^−^ (rate constant 1-2 × 10^10^ M/sec) [24] and in doing so enhances NO bioavailability and may enhance endothelium-dependent relaxation [25]. However, antioxidant capacity alone does not guarantee the ability to enhance endothelium-dependent relaxation. For example, the well-known antioxidant ascorbate (vitamin C) does not enhance endothelium-dependent relaxation in arteries when endogenous O_2_^−^ levels are enhanced by inhibiting SOD [10]. This is probably due to the relatively slow rate of reaction between ascorbate and O_2_^−^ (2 × 10^5^ M/sec) [26] since exogenous SOD did enhance relaxation. Therefore one of the aims of this study was to determine whether the tocotrienols scavenged O_2_^−^ rapidly enough to enhance endothelium-dependent relaxation in the presence of basal O_2_^−^ levels and when high concentrations of O_2_^−^ were generated by pyrogallol [27]. Surprisingly none of the tocotrienols were effective at improving endothelium-dependent relaxation, even at concentrations that decreased detection of superoxide generated by vascular tissue. By contrast, the less effective antioxidant *α*-tocopherol did improve ACh-induced relaxation. Significantly, tocomin was the compound that most effectively improved endothelium-dependent relaxation in the presence of pyrogallol-induced oxidative stress. These observations make an interesting comparison to reports that a tocotrienol rich extract was able to acutely improve impaired endothelium-dependent relaxation in aortae removed from spontaneously hypertensive rats or rats with type 1 diabetes caused by STZ [8]. A third component of tocomin, palm olein consisting mainly of triglycerides, was unlikely to account for the protective actions as it was reported to be without effect in the study by Muharis et al. [8].

Thus we speculated that the combination of multiple tocotrienol isomers and/or the additional presence of *α*-tocopherol was necessary to preserve endothelium-dependent relaxation. By testing the preparations with the same proportion of *α*-, *δ*-, and *γ*-tocotrienols and *α*-tocopherol present in tocomin, we determined that only the preparation containing *α*-tocopherol plus *α*-, *δ*-, *γ*-tocotrienol preserved endothelial function in the presence of oxidative stress. This data suggests an important interaction between *α*-tocopherol and tocotrienols to promote protection of vascular function. The mechanism of this positive interaction between *α*-tocopherol and the tocotrienols is worthy of further investigation.

We propose that the capacity of tocomin to preserve endothelium-dependent relaxation is by rapidly eliminating superoxide as has been previously reported with superoxide dismutase preservation of relaxation in the presence of oxidative stress [17]. Whilst tocopherols and tocotrienols have been reported to suppress signaling processes, for example, through the inhibition of NF-*κ*B and STAT [28], the rapid effect seen in this study seems more likely due to antioxidant activity.

## 5. Conclusion

It has been suggested that tocotrienols may have superior antioxidant activity to tocopherols, and we did find that to be true when superoxide is generated by hypoxanthine/xanthine oxidase* in vitro*. By contrast *α*-, *δ*-, and *γ*-tocotrienols were largely ineffective in improving NO mediated, endothelium-dependent relaxation in the presence of oxidative stress. However, tocomin, an extract from palm oil rich in tocotrienols and with a minor component of *α*-tocopherol, was found to be the most effective compound tested. The efficacy of tocomin could be replicated by the presence of *α*-tocopherol with *α*-, *δ*-, and *γ*-tocotrienols but not by the combined presence of the 3 tocotrienols alone. Thus the combination of tocotrienol isomers and tocopherol may prove to be an effective approach to the preservation of endothelial function where there is disease-induced oxidative stress such as in diabetes and hypertension.

## Figures and Tables

**Table 1:** The effect of tocomin, *α*-tocopherol (TC), and *α*-, *δ*-, and *γ*-tocotrienols (T3) on ACh-induced endothelium-dependent and SNP-induced endothelium-independent relaxation of rat aortae in the presence of pyrogallol-induced oxidative stress.

	*n*	ACh		SNP	
		pEC_50_ (M)	*R*_max⁡_ (%)	pEC_50_ (M)	*R*_max⁡_ (%)
*α*-tocopherol					
Control	5–7	−7.09 ± 0.18	95 ± 8	−8.71 ± 0.10	108 ± 7
Pyrogallol	5–7	−7.13 ± 0.18	47 ± 4^#^	−8.46 ± 0.14	97 ± 5
Pyrogallol + *α*-TC 10^−4^ mg/mL	5–7	−6.52 ± 0.21	47 ± 5^#^	−8.55 ± 0.12	94 ± 4
Pyrogallol + *α*-TC 10^−3^ mg/mL	5–7	−6.84 ± 0.12	46 ± 8^#^	−8.35 ± 0.28	96 ± 5
Pyrogallol + *α*-TC 10^−2^ mg/mL	5–7	−6.97 ± 0.15	70 ± 2^∗^	−8.57 ± 0.42	90 ± 5
*α*-tocotrienol					
Control	3–6	−7.08 ± 0.16	86 ± 10	−8.58 ± 0.25	97 ± 3
Pyrogallol	3–6	−6.97 ± 0.15	51 ± 2^#^	−8.67 ± 0.25	96 ± 7
Pyrogallol + *α*-T3 10^−3^ mg/mL	3–6	−6.25 ± 0.22	47 ± 4^#^	−8.41 ± 0.26	105 ± 4
Pyrogallol + *α*-T3 10^−2^ mg/mL	3–6	−6.58 ± 0.16	45 ± 5^#^	−8.40 ± 0.09	94 ± 7
Pyrogallol + *α*-T3 10^−1^ mg/mL	3–6	−6.82 ± 0.23	58 ± 5^#^	−8.12 ± 0.02	105 ± 4
*δ*-tocotrienol					
Control	3–6	−6.98 ± 0.12	80 ± 3	−8.38 ± 0.28	97 ± 4
Pyrogallol	3–6	−6.83 ± 0.45	42 ± 10^#^	−8.49 ± 0.20	99 ± 3
Pyrogallol + *δ*-T3 10^−3^ mg/mL	3–6	−6.74 ± 0.08	46 ± 9^#^	−8.73 ± 0.07	98 ± 2
Pyrogallol + *δ*-T3 10^−2^ mg/mL	3–6	−6.75 ± 0.29	43 ± 8^#^	−8.51 ± 0.05	95 ± 8
Pyrogallol + *δ*-T3 10^−1^ mg/mL	3–6	−6.64 ± 0.30	46 ± 8^#^	−8.60 ± 0.20	99 ± 2
*γ*-tocotrienol					
Control	3–6	−6.71 ± 0.24	93 ± 4	−8.50 ± 0.80	93 ± 4
Pyrogallol	3–6	−6.51 ± 0.15	59 ± 4^#^	−8.45 ± 0.20	98 ± 2
Pyrogallol + *γ*-T3 10^−3^ mg/mL	3–6	−6.16 ± 0.17	53 ± 4^#^	−8.34 ± 0.12	103 ± 1
Pyrogallol + *γ*-T3 10^−2^ mg/mL	3–6	−6.71 ± 0.27	55 ± 7^#^	−8.59 ± 0.15	95 ± 4
Pyrogallol + *γ*-T3 10^−1^ mg/mL	3–6	−6.65 ± 0.22	67 ± 1^#^	−8.68 ± 0.25	97 ± 1
Tocomin					
Control	5–7	−7.19 ± 0.08	92 ± 3	−8.69 ± 0.18	97 ± 4
Pyrogallol	5–7	−7.06 ± 0.13	58 ± 5^#^	−8.25 ± 0.05	99 ± 2
Pyrogallol + tocomin 10^−6^ mg/mL	5–7	−6.84 ± 0.14	63 ± 6^#^	−8.14 ± 0.24	100 ± 7
Pyrogallol + tocomin 10^−5^ mg/mL	5–7	−6.94 ± 0.13	69 ± 5^#^	−8.23 ± 0.18	103 ± 4
Pyrogallol + tocomin 10^−4^ mg/mL	5–7	−6.70 ± 0.20	81 ± 2^∗^	−8.17 ± 0.14	99 ± 2

^#^Significantly different to control *p* < 0.05.

^∗^Significantly different to pyrogallol *p* < 0.05.

Sidak's multiple comparison test.

**Table 2:** The effect of various combinations of *α*-tocopherol (TC) and *α*-, *δ*-, and *γ*-tocotrienols (T3) on ACh-induced endothelium-dependent and SNP-induced endothelium-independent relaxation of rat aortae in the presence of pyrogallol- (P-) induced oxidative stress.

	*n*	ACh		*n*	SNP	
		pEC_50_ (M)	*R*_max⁡_ (%)		pEC_50_ (M)	*R*_max⁡_ (%)
Treatment						
Control	6	−7.20 ± 0.15	93 ± 12	5	−8.68 ± 0.15	96 ± 2
Pyrogallol	5	−6.77 ± 0.07	50 ± 4^#^	5	−8.48 ± 0.09	100 ± 2
Pyrogallol + tocomin 10^−4^ mg/mL	5	−7.19 ± 0.13	80 ± 3^∗^	5	−8.34 ± 0.16	105 ± 5
P + T3 (*α* + *δ* + *γ*) + (*α*-TC) 10^−4^ mg/mL	5	−7.02 ± 0.11	73 ± 2^∗^	4	−8.49 ± 0.17	102 ± 2
P + T3 (*α* + *γ*) 10^−4^ mg/mL	6	−6.86 ± 0.29	65 ± 6^#^	5	−8.20 ± 0.21	102 ± 1
P + T3 (*α* + *δ* + *γ*) 10^−4^ mg/mL	3	−6.57 ± 0.23	61 ± 13^#^	5	−8.12 ± 0.08	103 ± 2

Tocotrienol isomers and *α*-tocopherol were present in the proportions found in tocomin (i.e., is *α*-T3- 20%, *δ*-T3 10%, and *γ*-T3 50% and *α*-TC 20%).

^#^Significantly different to control *p* < 0.05.

^∗^Significantly different to pyrogallol *p* < 0.05.

Sidak's multiple comparison test.
